# Chromatographic profile of major whey proteins in some dairy beverages based on milk serum

**DOI:** 10.55730/1300-0527.3497

**Published:** 2022-09-13

**Authors:** Doina PRODAN, Miuţa FILIP, Mihaela VLASSA, Marioara MOLDOVAN, Rahela CARPA

**Affiliations:** 1 Raluca Ripan Institute for Research in Chemistry, Babeş-Bolyai University, Cluj-Napoca, Romania; 2 Department of Molecular Biology and Biotechnology, Babeş-Bolyai University, Cluj-Napoca, Romania

**Keywords:** Dairy beverages, whey proteins, HPLC, validation

## Abstract

The aim of this study was the determination of chromatographic profiles of major whey proteins (WP): α-lactalbumin (α-La), β-lactoglobulin A and B, (β-Lg A and B), bovine serum albumin (BSA) in dairy beverages based on Zonar milk serum. The studied WP were separated by high-performance liquid chromatography (HPLC) on Aeris XB-C18 column using gradient elution with 0.1% trifluoroacetic acid (TFA) in water and 0.1% TFA in 80% acetonitrile, at 214 nm detection. The HPLC method was validated for system suitability, selectivity/specificity, linearity (R^2^ ≥ 0.996), precision (RSD% ≤ 2.01), trueness (recovery 96.29%–102.08%), sensitivity (limits of quantification (LOQ) 1.35–10.08 μg/mL), and robustness. The total of studied whey proteins (WPT) in dairy beverages based on Zonar milk serum varied between 1.42 g/L and 3.04 g/L. The results obtained were evaluated by principal component analysis (PCA) for correlation between the types of studied dairy beverages (natural and with additives added), the HPLC dataset of four whey proteins and WPT (active variables) to differentiate type of dairy beverages samples.

The obtained results confirm that the studied dairy beverages constitute a valuable source of bioactive components with benefits in human healthy nutrition and medical purposes.

## 1. Introduction

The milk whey represents a complex and heterogeneous mixture protein with wide biological, nutritional and technological applications in the formulation of modern food and beverages. Whey proteins are unique as they contain all the essential amino acids of good quality protein [1]. Bovine milk and colostrum are considered the most important sources of natural bioactive components, such as specific proteins, peptides, lipids, and carbohydrates [2]. In protein-containing foods such as milk and milk products, are found amino acids with a vital importance for human life. Eight amino acids are thought to be essential for humans, and they must be absorbed from foods containing animal proteins or a suitable combination of plant proteins, because the human body cannot synthesize them [3].

Considered an abundant dairy byproduct, the valorization of whey components is related to the recovery and concentration of whey proteins as new ingredients for food and nonfood sectors as well as the improvement of economic revenue for the dairy industry [4,5]. As dietary protein supplements the whey improve muscle strength and body composition, and can prevent cardiovascular disease and osteoporosis [2].

The whey proteins contain the major components β-lactoglobulin, α-lactalbumin, BSA and immunoglobulin and numerous minor proteins, such as lactoferrin, lactoperoxidase, proteose peptone, osteopontin, lysozyme, among others [6]. The characteristics and composition of milk whey depend on the source of milk (cow, sheep, goat, etc.), the feed of milk-producing animal, the stage of lactation, the processing method used and the time of the year when the milk samples were collected [7].

Recently, the researchers have developed a variety of methods to analyze whey proteins like as: reversed-phase high-performance liquid chromatography (RP-HPLC) [5,8–11], ultra-HPLC tandem mass spectrometry [10,12–16], electrophoretic techniques [10,17,18], immunoaffinity chromatography [19,20]. Enzyme-linked immunosorbent assay (ELISA) [21,22] and a novel visualized microarray method [23]. However, HPLC method allows the rapid and automated analysis, characterized by good separations, high resolutions and accuracy and reproducible results, especially for simultaneous detection of multiple whey proteins.

Whey protein is a source of α-lactalbumin, β-lactoglobulin, BSA, caseinomacropeptides, immunoglobulins, lactoferrin, lysozyme and offers health benefits, such as anticancer properties, enhanced immunity, antiviral, antimicrobial and antihypertensive properties. The conversion of whey into dairy beverages is one of the most attractive avenues for the utilization of whey for human consumption [24,25].

The milk whey is rich in protein residues, oligopeptides, amino acids and lactose [26,27], thus could be considered as an abundant resource to obtain antimicrobial peptides.

The purpose of this study was HPLC determination of major whey proteins (α-La, β-Lg A and β-Lg B, BSA) in some dairy beverages based on Zonar milk serum sweet whey as well as optimized and validating of the HPLC method. The results were evaluated by PCA for correlation between the types of studied dairy beverages (with and without additives added), the HPLC dataset of four WP (significant variables) and WPT, to differentiate type of dairy beverages samples.

Thus, the present study provides an important evaluation regarding valuable bioactive components, WP, in new experimental dairy beverages, in order to be used for nutritional and medical purposes.

## 2. Materials and methods

### 2.1. Reagents, standards and samples

All reagents were of analytical grade. The all standards of whey protein: α-(lactalbumin (α-La), β-lactoglobulin type A (β-Lg A), β-lactoglobulin type B (β-Lg B) and bovine serum albumin (BSA) were purchased from Sigma-Aldrich (Darmstadt, Germany). Ultrapure water (<18.3 MΩ cm) was prepared with Milli-Q Plus; Symplicity UV (Millipore SAS, Molsheim, France) water purification system (Merck KGaA, Darmstadt, Germany). Trifluoroacetic acid (TFA), acetonitrile (ACN) were purchased from Merck (Darmstadt, Germany).

The studied dairy beverages based on sweet whey (namely Zonar milk serum) obtained from cow milk were produced by SC EmbryOm Capital investment SRL (Satu Mare, Romania).

In this study, it was analyzed two types of Zonar milk serum dairy beverages. The first type includes seven Zonar milk serum dairy beverages without additives: six sample obtained from different milk batches (Z-1 to Z-6) and one sample a dairy beverage for baby children (Z-Baby). The second type includes three Zonar milk serum dairy beverages with additives added: a dairy beverage with natural extract of ginger extract and honey (5% aqueous extract) (Z-Gi-Ho); a dairy beverage with natural extract of cacao and honey (10% aqueous extract) (Z-Co-Ho); a dairy beverage with colloidal silver solution (15% of 1.5 ppm solution) (Z-Ag). Some physicochemical characteristics of some studied dairy beverages were presented in Table 1 [28].

The dairy beverages samples were kept at the fridge at 4 °C for at most one week, until performed all analysis.

### 2.2. HPLC method for WP determination

The analyses were carried out on a high-performance liquid Chromatography with UV/VIS detector (Jasco International Co., Ltd., Tokyo, Japan) and 20 μL sample loop (Rheodyne, Thermo Fischer Scientific, Waltham, MA, USA) for manual injection of sample. Separations were performed on a reversed-phase analytical column Aeris (WIDEPORE, 3.6 μm, XB-C18, 250 × 4.6 mm, 200 Å) column (Phenomenex); the system was controlled and the experimental data analyzed with the ChromPass software (version 1.7, Jasco International Co., Ltd., Tokyo, Japan). Mobile phase used was a mixture of two solvents by gradient elution. Solvent A consisted of 0.1% TFA in water and solvent B was 0.1% TFA in 80% ACN with the following gradient program: linear gradient from 35% B to 80% B for 15 min, from 80% B to 60% B for 5 min, and from 60% B to 35% B for 5 min; column temperature was kept to 40 °C, the flow rate at 0.7 mL/min and the detection wavelength was set at 214 nm. The injection volume consisted of 20 μL.

*Standard solution*. The stock standard solution of studied whey proteins (1 mg/mL each) was prepared in water. The stock solution was stored in the dark at 4 °C for no more than 1 month. Work standard solutions were prepared from stock solution and were diluted step-by-step with the water immediately before use.

S*ample preparation*. The dairy beverage sample (2 mL) was diluted with 3 mL 0.1% TFA in 5% ACN solution and 3 mL of 70% ACN solution. The final solution was centrifuged (Eppendorf 5804 R centrifuge, Hamburg, Germany) at 4500 rpm for 20 min at 20 °C and finally, the supernatant was passed through a 0.45-μm nylon membrane filter (Teknokroma, Barcelona, Spain) and injected in the HPLC system.

*Validation of the HPLC methodology*. The RP-HPLC method for the determination of WP in dairy beverages was validated for system suitability, selectivity/specificity, linearity, precision, trueness (recovery), sensitivity (limits of detection (LOD) and limits of quantification (LOQ)), and robustness according to ICH guidelines [29–33].

Suitability testing system was evaluated by parameters of chromatographic separation such as number of theoretical plates (N), retention factor (K), selectivity (α), resolution (Rs), peak symmetries, etc.

The selectivity of an analytical method is a measure which the method can determine simultaneously several components independently from each other without interference from matrix components. Specificity refers to the ability of the analytical method to differentiate and quantify the one analyte in the presence of other compounds that may be likely to be present.

Selectivity/specificity was tested by comparing the retention times (RT) of WP from chromatograms of standard solution and from Zonar milk serum dairy beverage and coelution assessment of studied WP with the other proteins peaks from studied beverage.

Linearity of the method was studied by injection of six known concentrations of standard solution of whey protein in the range of 20–100 μg/mL for BSA, 100–300 μg/mL for α-La, 50–250 μg/mL for β-Lg (A and B). Triplicate of 20 μL volume injections was made for each concentration. Four independent calibration curves were plotted by peak area versus concentration of the standard. Linear regression analysis was used to calculate the slope, intercept and correlation coefficient of each curve.

Sensitivity: The limit of detection (LOD) and limit of quantitation (LOQ) were calculated using signal-to-noise ratio of 3:1 and 10:1, respectively.

Precision: The analytical precision from the data of the intraday was obtained from 6 sample solutions at 100% of the test concentration and inject each. Interday precision was obtained from three concentrations and three replicates of each concentration for three consecutive days. These concentration levels represent about 80%, 100%, and 120% to the sample concentrations. The precision was expressed as a percentage of relative standard deviation (RSD %). The calculation of precision was based on the coefficient of variation (CV) according to Equation (1).


(1) Precision=100 [%]-CV

Trueness (or accuracy): To evaluate the accuracy of the method was studied the recovery degree. Standards addition was performed with preanalyzed standard solutions (three concentration and LOQ). Values derived from calibration standards at 80%, 100% and 120% of the sample concentration. Spiked samples were prepared in triplicate. The recovery was calculated in Equation (2) as follows:


(2) $$\text{Recovery }(\%)=\frac{Detected\;amount-Original\;amount}{Spiked\;amount}\times 100$$

Robustness: Robustness of the method was determined by observing the small changes in different experimental conditions, in the flow rate (± 0.1 mL/min), column temperature (± 2 °C) and wavelength (± 2 nm). The robustness of the method was evaluated from the RSD % of the peak area for each analyte after three consecutive injections of WP standard solution.

### 2.3. Statistical analysis

Statistical treatment, including calculation of mean, relative standard deviation, and standard error were performed with the Microsoft Excel software (version 2013, Microsoft Corporation, Redmond, WA, USA). Principal component analysis (PCA) was employed to evaluate the possible grouping of the dairy beverages, using the XLSTAT software, version 7.5.2, Addinsoft (Paris, France).

## 3. Results and discussion

### 3.1. HPLC method development and optimization

For separation of WP has been used a reversed phase column based on core-shell particle technology with large pore suitable for proteins separation. Based of literature data and characteristics of chromatographic columns we chose a specific column based on core-shell particle technology with great advantages in the separation of proteins and other large-molecular weight compounds [35,36]. Studies showed that particles with 160 Å pores allow full access of peptides and small proteins up to about 15 kDa without restricted diffusion, depending on solute configuration. Ostertag et al. (2021) tested several types of columns for protein separation, finally using a wide-pore column [8].

Small-diameter core-shell particles packed into columns can deliver a high level of performance, by an immediate improvement in analytical separations [37]. The Aeris reversed phase column with 3.6 μm widepore and 200 Å pore size provides efficient separation of whey proteins [35].

For optimization of HPLC separation were tested different mobile phase gradient programs based on two mobile phase solutions: 0.1% TFA in water (A) and 0.1% TFA in 80% ACN (B). The flow rate was tested between 0.4 mL/min and 0.9 mL/min, and a column temperature of 35 and 40 °C was applied. Therefore, the best HPLC conditions for whey proteins separation were linear gradient program, from 35% to 80% B in 15 min, from 80% to 60% B in 5 min, and from 60% to 35% B in 5 min; 40 °C column temperature; the flow rate at 0.7 mL/min and the wavelength of 214 nm.

Several studies reported the same detection conditions or the mobile phase eluent solutions, but various mobile phase gradient program [5,8,35]. The advantage of this method is that it uses the chromatographic column core-shell with higher resolution and highly reproducible separations of proteins and at low pH mobile phase.

### 3.2. HPLC method validation

According to the method validation guidelines [29–31], system suitability tests are an integral part of an LC method and are used to verify that the column efficiency (N), selectivity factor (resolution) and reproducibility of the chromatographic system are adequate for the analysis. In our case, the system suitability test was carried out on freshly prepared standard stock solutions of BSA, α-La, β-Lg B and β-Lg A and the parameters are shown in Table 2. The system suitability test was found to be suitable.

The analytical performance of the present method must be evaluated by the selectivity/specificity, linearity, sensitivity, precision, trueness (recovery), LOD, LOQ, robustness [29,33] in order to ensure that the method is suitable for its intended use.

**Figure 1:**
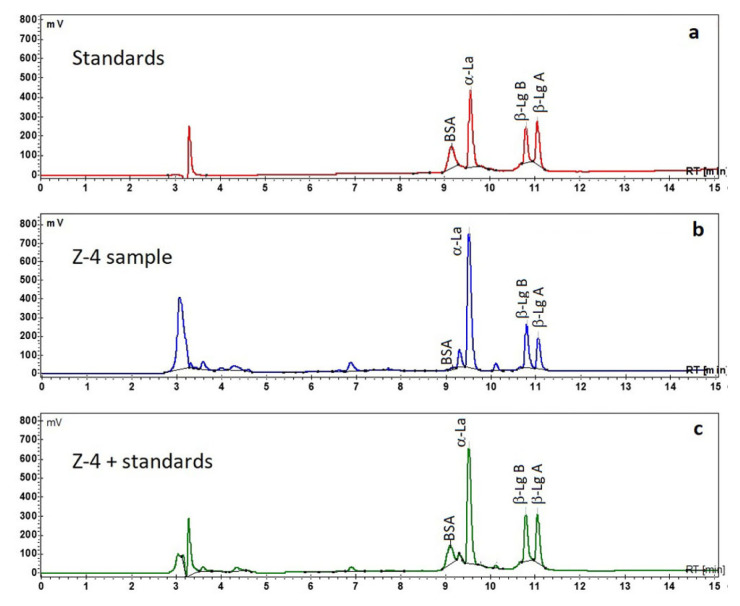
Chromatograms of: (a) standard solution of WP (each of 150 μg/mL), (b) Zonar milk serum dairy beverage (Z-4) and (c) Zonar milk serum dairy beverage (Z-4) with addition of WP standard mixture (each of 250 μg/mL) in ratio 1:1, (v/v).

The specificity (Figure 1) was evaluated by comparing the chromatograms of the protein standard solution (each WP of 150 μg/mL, Figure 1a), Zonar milk serum dairy beverage (Z-4) (Figure 1b) and Z-4 sample with standards solution added (each WP of 250 μg/mL) in ratio 1:1 (v/v) (Figure 1c). There was no coelution of studied WP with the other proteins peaks from studied beverage.

The elution order (Figure 1a) shows that BSA and α-La protein were eluted before the β-lactoglobulins components (β-Lg A and B). Also, the β-Lg B protein was eluted before β-Lg A protein [11,14,35].

In chromatograms presented in Figures 1b and 1c, a good separation of each studied proteins can be observed. Also, the small peaks (e.g., at RT at 6.9, 9.4, and 10.2 min) are attributable to the other proteins, like lactoferrin, immunoglobulins and lactoperoxidase [8,14] present in Zonar milk serum sample. Also, to verify separation selectivity we observed that no coelution of other small proteins with the studied WPs was detected (Figure 1c). Thus, it can be concluded that the proposed HPLC method was a selective one.

Linearity of the method, evaluated through the construction of four calibration curves, was based on the external standard method at six different concentration levels. The calibration curves were plotted by the peak area versus concentration of each studied WP. Results show linear relationship in the range of 20–100 μg/mL for BSA, 100–300 μg/mL for α-La and 50–250 μg/mL for β-Lg (A and B). This linearity range covers the protein quantities that are found in the studied samples. Parameters of calibration curves are reported in Table 3.

As seen in Table 3, the good regression coefficients, R^2^ > 0.996 of calibration curves were obtained within test ranges. The LODs and LOQs were found in the range of 0.4–3.2 μg/mL and 1.35–10.08 μg/mL, respectively, showing a good sensitivity of the method. Ren et al. (2010) obtained a lower limit of quantitation of 0.15–0.19 μg/mL for bovine proteins α-La and β-Lg in infant formulae [12].

Intra- and interday precision for each whey protein at different concentration levels are listed in Table 4. The concentration levels for intraday precision are close with the real measured concentrations at LOQ level. The amounts represent of the standard solutions of approximately 100% of each protein found in the milk serum sample. The calculated RSD values were found to be small, below 2%, indicating good repeatability and reliability of the proposed method.

The intraday precision (RSD %) varied from 0.61 to 1.80 while the interday precision ranged varied from 0.44 to 2.01. Results indicate that the method for quantification of whey protein is precise confirmation validation guidelines [29,30].

Trueness (accuracy) of the measurements was determined using the calibration standards of proteins at three concentration and LOQ each, in triplicate, and assessed by the recovery of added standard (Table 5) to Zonar milk serum sample (Z-4). WP concentration derive from calibration standards at 80%, 100%, and 120% of the sample concentration.

The obtained recoveries of WP of Zonar milk serum sample (Z-4) were from 96.29% to 102.08%. Thus, it can be concluded that the validated HPLC method can be used in laboratory for routine analysis of whey proteins from milk serum beverages.

HPLC methods for protein determinations from different matrices were validated by some authors obtaining similar results [10,16,38].

In Table 6, the results of robustness testing showed acceptable limits (RSD less than 2.0%), for a minor change of method conditions, such as the flow rate, column temperature and wavelength [32,33]. Thus, the HPLC method is robust.

### 3.3. HPLC determination of WP in dairy beverages

**Figure 2:**
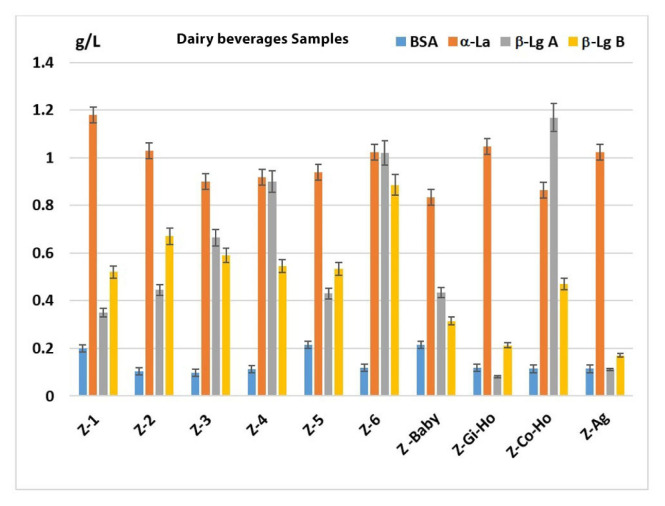
The major whey proteins (BSA, α-La, β-Lg A and β-Lg B) concentrations (mean ± SEM) in Zonar milk serum dairy beverages.

The validated HPLC method for determination of WPs was applied to analysis of ten Zonar milk serum dairy beverages. The results are presented in Figure 2.

The results show that the α-La is major whey protein in all these dairy beverages, with amounts (g/L) between 1.18 (Z-1) and 0.83 (Z-Baby). Also, the amounts of BSA show smaller values (g/L) between 0.10 (Z-2) and 0.22 (Z-1). The β-Lg A was found in a larger quantity (g/L) in studied beverages with values of 0.90 (Z-4), 1.01 (Z-6), and 1.16 (Z-Co-Ho), respectively. The β-Lg B whey protein was found in amounts (g/L) between 0.31 (Z-Baby) and 0.88 (Z-6), for natural milk serum dairy beverages, and between 0.17 (Z-Ag) and 0.47 (Z-Co-Ho) for dairy beverages with additives.

Instead, the total of studied major whey proteins (WPT) in these dairy beverages varied between 1.42 g/L and 3.04 g/L, similar results were obtained by Sturaro et al. [5].

The contents of the α-La, β-Lg A, and β-Lg B in bovine colostrum and bovine raw milk were reported in infant formula, with values between 2 g/L and 2.8 g/L [10]. Also, the β-Lg levels in 71 different Austrian retail milk samples were obtained in amounts ranging from around 0.1–4 g/L [14]. Rotkāja et al. (2016) determined the α-La and total β-Lg in milk samples with values in the ranges of 0.8–1.5 g/L and 0.094–2.6 g/L, respectively [11].

The composition and characteristics of whey are depending on the milk source, the feed of the milk-producing animal, the processing method used, the time of the year, and the stage of lactation [7].

The whey proteins are the valuable constituents of sweet whey and stand out for their high nutritional value in terms of biological value and composition in essential amino acids. α-La is a primary protein found in human breast milk and together with β-Lg are a source of essential and branched chain amino acids. Besides, the BSA is also a source of essential amino acids [2,39].

### 3.4. Principal component analysis (PCA)

**Figure 3:**
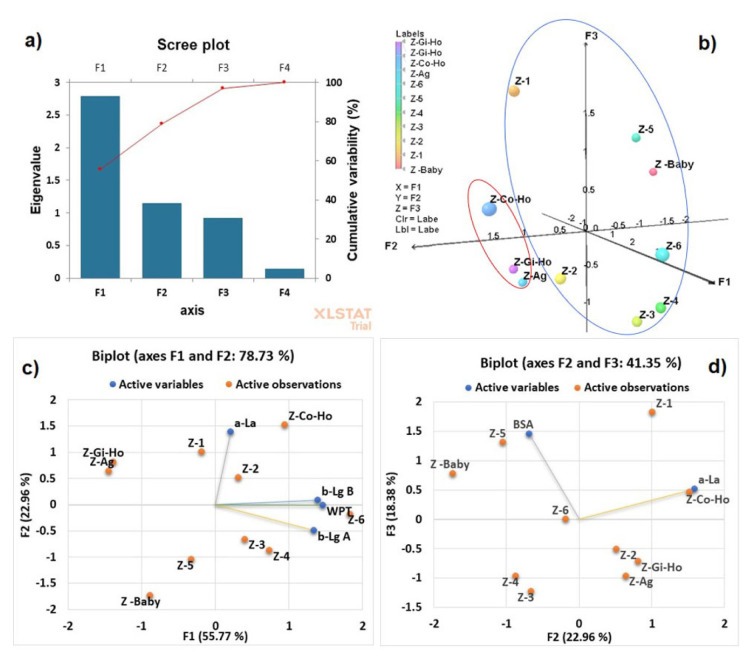
PCA results: a) eigenvalues and cumulative variability (%), b) 3D plot of dairy beverages samples: with additive added (marked red) and without additive (marked blue), c) biplot PCA with F1 and F2 factors, and d) biplot PCA with F2 and F3 factors, loadings of whey proteins and WPT (active variables) and dairy beverages samples (active observations).

Principal component analysis (Figure 3) was performed for an exploratory evaluation involving 10 dairy beverages (active observations) and the dataset of four whey proteins and WPT (active variables) obtained by HPLC to differentiate type of dairy beverages samples.

The first two principal components, PC1 (F1) and PC2 (F2), accounted for 78.73% of the total variance (55.77% for PC1 and 22.96% for PC2), and provided discriminatory information related to the samples.

The eigenvalues of the correlation matrix for PC1, PC2, PC3 (F3), and PC4 (F4) were, respectively, 2.788, 1.148, 0.919, and 0.144 (Figure 3a). Thus, the principal components showed separation mainly into two groups in 3D plot (Figure 3b), according to the types of studied dairy beverages: with additives added (marked red) and without additives added (marked blue). Figure 3c shows the score plot (F1, F2) of PCA for different dairy beverages samples in relation to individual whey proteins and total whey proteins obtained by HPLC. The projection of the dairy beverage samples on biplots with F1, F2 and F2, F3 factors (Figures 3c and 3d) shows the increase of the scores at 78.73% and 41.35%, respectively.

Principal component analysis (PCA) is a widely used multivariate statistical analytical technique. Thus, the profiling specific product characteristic, comparing of similar products based on imperative attributes to consumers and thus increasing market share by altering product characteristics are some of the fates of PCA results [40]. PCA has been used to characterize sensorial attributes with a great degree of success in many food products, e.g., beverages formulated with whey protein [40], ultrapasteurized milk [41], fermented food products [42], yoghurt [43].

Antimicrobial and antiviral actions, immune system stimulation, anticarcinogenic activity and other metabolic features have indeed been associated with such whey proteins, α-lactalbumin, β-lactoglobulin, lactoferrin, lactoperoxidase, and bovine serum albumin [44].

Bioactive components in α-La and β-Lg derived peptides are helpful to protect against hypertension through ACE-inhibitory activity and to regulate blood pressure [26,39]. The β-Lg protein has the role in resistant carrier of retinol (a provitamin A) and participates in the digestion of milk lipids during the neonatal period, and furthermore, this protein may play a role in the absorption and subsequent metabolism of fatty acids [45].

These Zonar milk serum dairy beverages have similar physicochemical characteristics with the sweet whey. The low total protein and mineral content and the moderate quantity of lactose [28] are recommended for curative and palliative purposes in medical fields such as obesity, diabetes mellitus type II or kidney diseases.

## 4. Conclusion

In this study, the determination of major whey protein in Zonar milk serum dairy beverages as bioactive compounds was performed. A simple and efficient RP-HPLC method was successfully optimized, validated and applied for determination of whey protein (BSA, α-La, β-Lg A and B) in Zonar milk serum dairy beverages providing satisfactory trueness with low limits of detection. The total studied WP in dairy beverages varied between 1.42 g/L and 3.04 g/L.

The projection of the dairy beverage samples with PCA evaluated by HPLC dataset of four whey proteins and WPT (active variables) showed that they are separated mainly into two groups according to the types of studied dairy beverages (with and without additives added).

The obtained results confirm that Zonar milk serum dairy beverages constitutes a valuable source of bioactive components which are recommended for human healthy nutrition, as well as for curative and palliative purposes in medical fields such as obesity, diabetes mellitus type II or kidney diseases.

## Figures and Tables

**Table 1:** Physicochemical characteristics of studied dairy beverages based on Zonar milk serum [28], average nutritional values/100 mL of product.

Characteristics	Z-4	Z-Gi-Ho	Z-Co-Ho	Z-Ag
pH	6.2	6.1	6.2	6.3
Acidities (°T)	9.0	10.0	10.9	9.9
Dry matter (%)	6.3	6.1	6.1	6.2
Water (%)	93.7	93.9	93.9	93.8
NaCl (%)	0.2	0.19	0.19	0.19
Lactose (%)	4.8	4.7	4.7	4.6
Energetic value	30 kcal			
Fats	0.1 g			
of which saturated fatty acids	0.05 g			
Carbohydrates	4.82 g			
of which sugars	0 g			
Proteins	0.8–0.9 g			
Salt	0.52 g*			

* Sodium equivalent naturally present in milk serum; Z-4, Zonar milk serum sample.

**Table 2:** Analytical parameters for system suitability test of HPLC method.

Parameter	BSA	α-La	β-Lg B	Β-Lg A	Reference values [30–33]
Retention time [RT, min]	9.14	9.56	10.80	11.05	
Peak width at base (Wb)	0.27	0.15	0.12	0.14	
Dead time [T_M_, min]	3.28				
Selectivity [α]	1.12	1.07	1.19	1.03	>1
Retention factor [K]	1.78	0.65	0.69	0.70	0.1–10 acceptable
Resolution [Rs]	2.73	1.95	8.79	1.845	Rs > 2 [33] Rs >1.5 [30]
Plate number [N]	17934.40	60087.83	117551.02	92919.85	> 2000*
Symmetry factor	1.22	1.66	1.66	1	≤2
HETP [cm]	0.0013	0.00041	0.00021	0.00026	L/N small

HETP, height equivalent theoretical plates [cm]; L, column length;

* increases with efficiency of the separation; L/N, the smaller the value, the higher the column efficiency.

**Table 3:** Validation parameters for HPLC-UV method optimized for the determination of whey proteins in studied dairy beverages.

Parameters	BSA	α-La	β-Lg A	β-Lg B
Retention time, RT (min)	9.12	9.56	10.79	11.05
Linear range (μg/mL)	20–100	100–300	50–250	20–250
Calibration curve	A = 0.18371·C + 3.1489	A = 0.21711·C + 0.5138	A = 0.12492·C + 0.3146	A = 0.14051·C + 0.2056
Regression coefficient *R^2^* (n = 6, six points)	0.9967	0.9994	0.9979	0.9988
LOD (3 × S/N, μg/mL) ± SD	3.24 ± 0.82	0.40 ± 0.24	0.75 ± 0.46	0.64 ± 0.48
LOQ (10 × S/N, μg/mL) ± SD	10.08 ± 1.09	1.35 ± 0.71	2.50 ± 0.65	2.14 ± 0.59

S/N, signal to noise ratio; A, peak area; C, concentration of analyte (μg/mL); RSD, relative standard deviation; SD, standard deviation.

**Table 4:** Evaluation of the precision of the proposed HPLC method.

Intraassay precision, n = 6 (replicates) + LOQ				
Protein	Concentration (μg/mL)	Mean %	SD	RSD %
BSA	*10	96.69	1.09	1.12
	50	97.5	1.76	1.80
α-La	*1.35	98.31	0.710	0.72
	250	100.52	0.623	0.62
β-Lg B	*2.5	97.81	0.650	0.66
	160	101.08	1.748	1.73
β-Lg A	*2.15	97.27	0.590	0.61
	110	98.75	1.036	1.05
Interassay precision, n = 9 (3 concentration / 3 replicates) + LOQ				
BSA	*10	96.15	1.12	1.16
	40	97.84	1.94	1.98
	50	100.72	1.24	1.23
	60	101.45	1.05	1.03
α-La	*1.35	97.04	1.04	1.07
	200	99.79	0.89	0.89
	250	101.16	0.45	0.44
	300	102.24	1.04	1.02
β-Lg B	*2.5	96.97	1.57	1.62
	130	100.81	0.99	0.98
	160	98.04	1.97	2.01
	200	100.32	0.98	0.98
β-Lg A	*2.15	98.27	0.96	0.98
	90	98.64	1.95	1.98
	110	101.23	1.06	1.05
	130	102.04	1.84	1.80

RSD, relative standard deviation; SD, standard deviation.

* LOQ, values derived from calibration standards at 80%, 100% and 120% of the sample concentration. Intraassay precision, n = 6 (replicates) and LOQ. Interassay precision, n = 9 (3 concentrations / 3 replicates each, 3 consecutive days) and LOQ.

**Table 5:** Evaluation of the trueness (accuracy) of the proposed HPLC method.

Proteins	Initial μg/mL	Taken μg/mL	Found μg/mL	Recovery ( %)	SD	RSD %
BSA	52.40	*10	62.03	96.29	1.58	1.64
		40	91.64	98.11	2.02	2.06
		50	103.24	101.68	1.69	1.66
		60	113.62	102.04	1.51	1.48
α-La	257.00	*1.35	258.32	97.56	1.76	1.80
		200	458.96	100.98	1.54	1.53
		250	504.93	99.17	1.31	1.32
		300	563.24	102.08	1.58	1.55
β-Lg B	167.60	*2.5	170.03	97.38	1.82	1.87
		130	299.23	101.25	1.41	1.39
		160	325.97	98.96	1.99	2.01
		200	364.34	98.37	1.29	1.31
β-Lg A	111.27	*2.15	113.37	97.84	1.51	1.54
		90	200.43	99.07	2.04	2.06
		110	223.02	101.59	1.79	1.76
		130	239.36	98.53	1.98	2.01

RSD, relative standard deviation; RSD (%), relative standard deviation; SD, standard deviation.

* LOQ values; WP concentration derived from calibration standards at 80%, 100%, and 120% of the sample concentration.

**Table 6:** Evaluation of the robustness of the proposed HPLC method.

Robustness (RSD %)			
Parameters	Flow rate (± 0.1 mL/min)	Column temperature (± 2 °C)	Wavelength (± 2 nm)
BSA	1.83	1.92	1.22
α-La	0.93	0.39	0.25
β-Lg B	1.51	1.08	0.49
β-Lg A	1.23	0.86	0.57

RSD %, relative standard deviation.
